# Spatiotemporal Gait Analysis and Lower Limb Functioning in Foot Dystonia Treated with Botulinum Toxin

**DOI:** 10.3390/toxins10120532

**Published:** 2018-12-12

**Authors:** Anupam Datta Gupta, Graeme Tucker, Simon Koblar, Renuka Visvanathan, Ian D. Cameron

**Affiliations:** 1 Rehabilitation Medicine, The Queen Elizabeth Hospital, 28 Woodville Road, Adelaide 5011, Australia; 2 Discipline of Medicine, University of Adelaide, Adelaide 5005, Australia; 3 National Health and Medical Research Council Centre of Research Excellence in Frailty and Healthy Aging, University of Adelaide, Adelaide 5005, Australia; grtucker@adam.com.au; 4 Director Stroke Research Programme, South Australian Health & Medical Research Institute (SAHMRI), GPO Box 11060, Adelaide 5001, Australia; Simon.Koblar@adelaide.edu.au; 5 Director Aged and Extended Care, The Queen Elizabeth Hospital, 28 Woodville Road, Adelaide 5011, Australia; Renuka.Visvanathan@adelaide.edu.au; 6 Head John Walsh Centre for Rehabilitation Research, Sydney Medical School, University of Sydney, Sydney 2006, Australia; Ian.Cameron@sydney.edu.au

**Keywords:** botulinum toxin, foot dystonia, gait analysis

## Abstract

Foot dystonia (FD) is a disabling condition causing pain, spasm and difficulty in walking. We treated fourteen (14) adult patients experiencing FD with onabotulinum toxin A injection into the dystonic foot muscles. We analyzed the spatiotemporal gait utilizing the GaitRite system pre- and 3 weeks post-botulinum toxin injection along with measuring dystonia by the Fahn–Marsden Dystonia Scale (FMDS), pain by the Visual Analog Scale (VAS) and other lower limb functional outcomes such as gait velocity, the Berg Balance Scale (BBS), the Unified Parkinson’s Disease Rating Scale–Lower Limb Score (UPDRS–LL), the Timed Up and Go (TUG) test and the Goal Attainment Scale (GAS). We found that stride length increased significantly in both the affected (*p* = 0.02) and unaffected leg (*p* = 0.01) after treatment, and the improvement in stride length was roughly the same in each leg. Similar results were found for step length (*p* = 0.02) with improvement in the step length differential (*p* = 0.01). The improvements in the lower limb functional outcomes were also significant—FMDS, VAS, TUG, and UPDRS–LL decreased significantly after treatment (all *p* < 0.001), and BBS (*p* = 0.001), GAS (*p* < 0.001) except cadence (*p* = 0.37). BT injection improved walking in foot dystonia as evidenced through gait analysis, pain and lower limb functional outcomes. Main study limitations were small sample size and lack of control.

## 1. Introduction

Dystonia is a movement disorder characterised by sustained or intermittent muscle contractions causing abnormal, often repetitive movements, postures, or both. Dystonic movements are typically patterned, twisting, and may be tremulous. Dystonia is often initiated or worsened by voluntary actions and associated with overflow muscle activation [1].

Focal dystonia can affect any part of the body such as jaw muscles (oromandibular dystonia), cervical region (torticollis), periorbital muscles (blepharospasms) and upper limb dystonia known as writer’s cramp. Foot dystonia (FD) can occur in Parkinson’s disease (PD), may be associated with spasticity, and can occur in isolation. Task-specific lower limb dystonia such as runners’ dystonia is also reported. When it occurs, FD can have significant functional implications. Individuals experience pain, spasm and difficulty wearing shoes. The dystonic foot does not come fully in contact with ground and impairs balance during the stance phase (both the single and the double support phases) of gait. This causes difficulty in walking and in some cases, falls and fractures can occur [2]. Activities of daily living and recreational activities are adversely affected with consequential poor quality of life. Walking is an important human activity, essential for independence, working and participation in different societal activities. Walking is a great way of maintaining and improving heath, now considered an important vital sign [3]. Several meta-analysis studies make a strong case for walking in preventing cardiovascular events, type 2 diabetes, osteoporosis and stroke to name a few. Reduced walking is associated with poor health outcome, institutionalisation and even mortality [4].

Despite the above, there is limited literature on FD. Additionally, FD is poorly characterised in comparison to other focal dystonia. The treatment of FD is usually multimodal such as medications, physiotherapy and orthotic devices. FD can be refractory, as the medications used are often ineffective and can cause adverse effects. The most effective treatment for any focal dystonia is botulinum toxin (BT) injection [5]. The toxin reversibly blocks acetylcholine attachment to the post synaptic vesicles. The onset of action is 7–10 days and the treatment effect usually can last for 3–4 months, and patients may need repeat injections after the effects of the toxin has worn off. Dystonia is normally assessed by the Fahn–Marsden Dystonia Scale (FMDS) rating scale—a subjective ordinal scale.

Objective gait analysis is emerging as an important way of evaluating lower limb function such as walking in many neurological disorders. Although there are papers on gait analysis in different neurological conditions such as Parkinson’s disease (PD) and stroke, including research from our group [6], the authors could not identify papers relating to gait analysis in FD. The GaitRite system (instrumented walkway) is a well-studied and validated method of evaluating gait [7]. This system studies the kinematic aspects of gait including the spatiotemporal parameters (STP) such as step length, step time, single support, double support (spatial) and temporal parameters such as step time, stride time, single support, double support, step length differential etc. The system also generates gait velocity, cadence and the foot fall parameters allowing objective measurements of gait. Following an intervention such as botulinum toxin injection, gait analysis may help clinicians to objectively evaluate the response and so record outcomes more objectively.

The aim of this pilot study was to evaluate the spatiotemporal gait parameters and to note changes in those parameters following BT injection along with dystonia, pain and the lower limb functional parameters such as balance, Timed Up and Go (TUG), Unified Parkinson’s Disease Rating Scale–lower limb (UPDRS–LL) score, Goal Attainment Scale (GAS) goals in 14 individuals with FD from different aetiologies.

## 2. Results

The demographic details such as age, height, weight of the participants along with the diagnosis, muscles involved, and dosing of BT are shown in Table 1.

**Figure 1:**
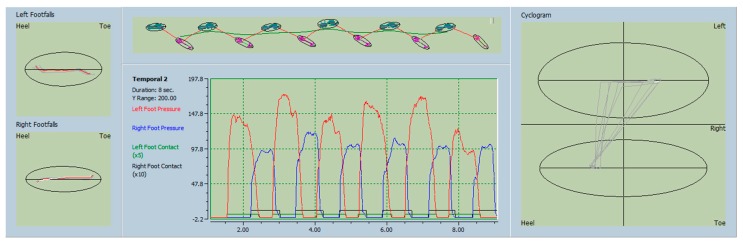
Pre-injection gait analysis—gait analysis in a patient with left foot dystonia (FD). Pre-injection gait analysis showing significant asymmetry of right and left foot pressures and asymmetric cyclogram i.e., propulsion of the body.

**Figure 2:**
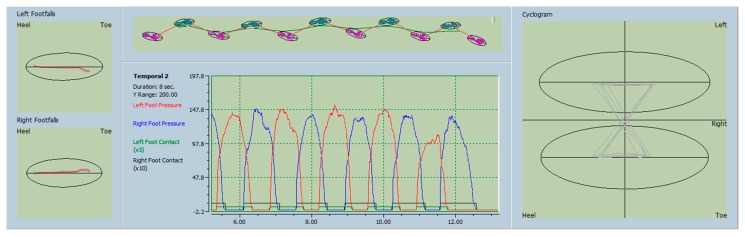
Post-injection gait analysis—gait analysis post-BT injection shows significant restoration of the asymmetry of right and left foot pressures and a symmetric cyclogram (analyzed by Protokinetics software).

The changes in the STP of gait were quite discernable from the pre- and post-intervention recording of the gait using the updated software (Figure 1 and Figure 2).

**Figure 3:**
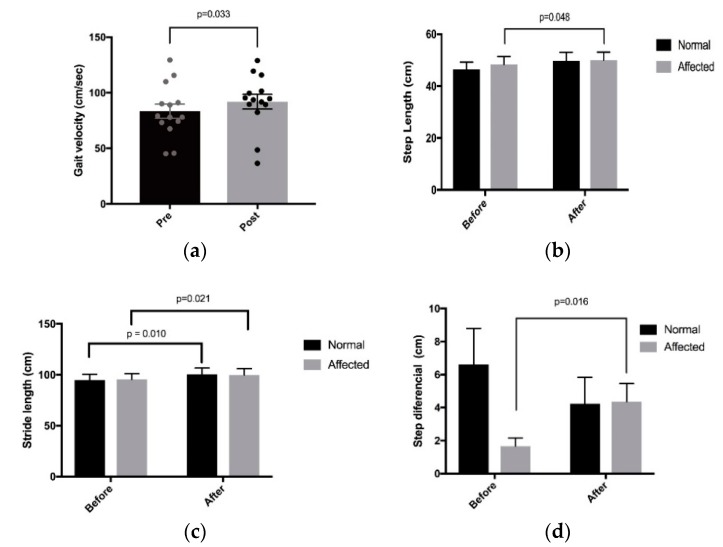
Bar Graphs—Gait parameters—Gait Velocity, Step Length, Stride Length, Step Differential. (**a**): Gait velocity; (**b**): Step length; (**c**): Stride length (**d**): Step differential.

The comparisons over time detailed here are adjusted for age, gender, and side in panel data repeated measures models. We found that (Figure 3) stride length increased significantly in both the affected (*p* = 0.021) and unaffected (*p* = 0.010) legs after treatment, and that the improvement in stride length was roughly the same in each leg (*p* = 0.079). Step length increased in the abnormal leg (*p* = 0.048). The increase in step length for the normal leg was marginally significant (*p* = 0.070). The change in step length for the normal leg was not significantly different to the change in step length for the abnormal leg (*p* = 0.525). The increase in step differential observed for the abnormal leg was significant (*p* = 0.016). The decrease in step differential observed for the normal leg was not significant (*p* = 0.444). The change in step differential for the normal leg was not significantly different to the change in step differential for the abnormal leg (*p* = 0.128). No significant differences were found for step time, cycle time, swing time, stance time, single support, or in double support. There were no significant changes in symmetry ratio over time for step length (*p* = 0.971), swing time (*p* = 0.687), or stance time (*p* = 0.613). We have included the standard error (SE) in Table 2 rather than the standard deviation as the standard error is the standard deviation divided by the square root of the sample size, and is the basic quantity determining the width of the confidence interval.

**Figure 4:**
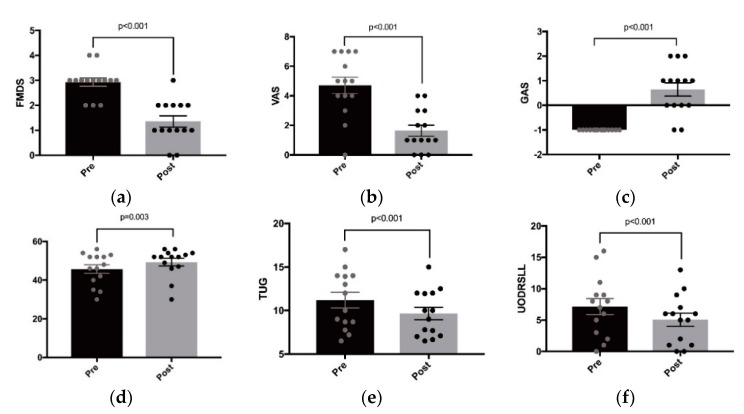
Bar Graphs—other lower limb functional parameters—dystonia (Fahn–Marsden Dystonia Scale (FMDS)), pain (Visual Analog Scale (VAS), Goal Attainment Scale (GAS)), balance (Berg Balance Scale (BBS), Timed Up and Go (TUG), Unified Parkinson’s Disease Rating Scale–lower limb (UPDRS–LL). (**a**): FMDS; (**b**): VAS; (**c**): GAS; (**d**): BBS (**e**): TUG; (**f**): UPDRS-LL.

For the FMDS, Visual Analog Scale (VAS), Berg Balance Scale (BBS), UPDRS–LL, cadence and gait velocity, we have before and after measures, but these were not related to any specific leg, unlike the other gait parameters analyzed previously. We found that allowing for sex, age and side of abnormality, FMDS, VAS, TUG, and UPDRS–LL decreased significantly after treatment (all *p* < 0.001), and BBS (*p* = 0.001), GAS (*p* < 0.001), and gait velocity (*p* = 0.028) increased significantly (Figure 4). No difference between before and after measurements was detected for cadence (*p* = 0.376).

Amongst the 14 patients, two patients did not respond to the injection of botulinum toxin with regards to their FD. This might be ascribed to the advanced nature of their underlying Parkinson’s disease. The injection of BT was well tolerated and none of the patients experienced any adverse events.

## 3. Discussion

In this study we found that BT improves some STP parameters of gait and gait velocity along with other important lower limb functional parameters such as balance (Berg Balance Scale), mobility (Timed Up and Go), patient reported goals (GAS/Goal Attainment Scale) and gait velocity. There was a significant improvement in dystonia (FMDS), pain (VAS) and the lower limb score of the Unified Parkinson’s Disease Rating Scale.

Amongst the STP of gait, we found individuals with FD improved with their stride length and step length. Shorter stride and step length were noted in both affected and unaffected leg pre-injection. Following BT injection, the stride length increased in both legs significantly (*p* < 0.021), The step length was also increased (*p* < 0.048), in the affected and unaffected leg although the change was marginal in the normal leg following the injection of BT. The improvements can be explained by the observation that individuals could place the foot better after dystonia resolved with the BT injection, as the foot was coming more in contact with the ground with normal weight transfer. The changes could be explained by the concept that gait is a bipedal activity; if one leg is affected, the other tries to compensate by adjusting to the abnormal side. In this study we did not notice any significant changes in the temporal parameters of gait such as stride time, step time, single or double support and in cadence.

As mentioned earlier, walking is an important activity for the maintenance of health, independence and quality of life. It is also considered as one of their important goals by the patients with neurological impairments [8]. Moreover, walking (gait) is increasingly considered as an important functional outcome measure in rehabilitation research. Gait is primarily assessed through gait velocity. In this series, patients improved significantly with their gait velocity. Beyond the gait velocity, we also looked at other important gait parameters as described above. Recent research showed that gait velocity may not represent or capture the impairments in walking [9]. For example, from the clinical experience we have seen that the patients with PD or FD can walk with reasonable speed but that does not mean that their walking was normal. Above and beyond these parameters, new insight into human walking in neurological conditions suggest other aspects of gait such as symmetry [10] and variability [11,12] should be measured. We did not notice any significant change in the gait symmetry in this group of patients. We could not assess the gait variability because we did not have data for the standard deviation of swing time, single support, double support, or gait velocity, so the formula could not be applied.

Despite this, the study underpins that it is possible to show changes in the STP of gait along with several other lower limb functional parameters in patients with FD following BT injection.

All patients in the study group complained about significant foot pain during walking along with dystonia. We looked at balance by BBS as balance is integral to the lower limb functioning (as the patient must balance on one leg for standing and walking). TUG is considered an important test for mobility and has significant inter-rater reliability in predicting falls. Many patients with FD have a propensity towards falls and suffering adverse consequences such as fracture [2]. We have also calculated the lower limb parameters of the UPDRS scale and patient reported goals i.e., GAS goals which are increasingly considered important outcomes in rehabilitation research [13]. We found a significant reduction in pain besides the reduction in dystonia and improvement of other lower limb functional parameters with BT injection. The toxin was effective for 4–5 months and all the patients returned for further injections.

In rehabilitation, it is often difficult to show the improvement in objective functional parameters. We have attempted to show that improvement in gait and LL functional improvement can be shown in this group of patients with FD following BT injection. A randomized controlled study (RCT) has recently been published since we published our pilot study on the effects on BT injection a group of PD patients with FD who had deep brain stimulation [14]. In this extended report we found how gait parameters along with other lower limb functional parameters can be used to evaluate function following BT injection. This we hope will pave the ways for further studies with functional outcomes leading to the health administrators considering providing BT at subsidised cost to this group of patients. BT is still not approved by the Pharmaceutical Benefit Scheme in FD in many countries.

## 4. Study Limitations

The major limitation of this pilot study is that it is uncontrolled with a small sample size. We did not measure quality of life pre- and post-intervention. Despite this, the study provided useful insight in evaluating effectiveness of BT injection in terms of functional improvement.

## 5. Conclusions

It is important to show functional improvement following any therapeutic intervention in patients with neurological disorders. The evidence base of lower limb functional improvement following botulinum toxin injection for spasticity or dystonia is not robust. This study showed significant reduction in dystonia, improvement in pain and gait parameters, along with other lower limb functional parameters such as balance (BBG), mobility (TUG), and patient-reported outcome (GAS) following injection of botulinum toxin in FD. Randomized controlled studies should be undertaken for proving the efficacy of BT in improving the functional outcomes in FD.

## 6. Methods

### 6.1. Design

Pre- and post-treatment comparison. Open-label observational study.

### 6.2. Ethics

The Hospital Research Ethics Committee approved the study and all the patients provided written informed consent (Central Adelaide Local Health Network (CALHN) reference number: Q20180815, date of approval—27 August 2018).

### 6.3. Participants

Fourteen (14) adult patients, men and women over 40 years of age with the diagnosis of Parkinson’s disease in 11 patients and 3 patients without the diagnosis of any underlying definitive neurological conditions but experiencing intractable foot dystonia participated in the study. We included adult patients with foot dystonia who were independently mobile without any assistive device or mobility aids. Non-ambulatory patients with FD were excluded from the study. All the patients had unilateral foot dystonia affecting their walking. Patients were recruited as they were available (convenience sampling).

### 6.4. Setting

The patients were assessed in the multidisciplinary spasticity clinic which consisted of the rehabilitation physician, medical registrar, nurse, physiotherapist, occupational therapist, and orthotist at the Queen Elizabeth Hospital.

### 6.5. Physical Assessments

Patients were assessed in the outpatient clinic by the rehabilitation physician and the multidisciplinary team pre- and 3 weeks post-injection of botulinum toxin. After collecting the basic demographic data, height, weight the following outcome measures were noted—spatiotemporal gait analysis and gait velocity (primary outcomes), the secondary outcomes were—FMDS, pain score by Visual Analog Scale during walking, balance by Berg Balance Scale, Unified Parkinson’s Disease Scale for lower limbs, Timed Up and Go tests, and Goal Attainment Scale.

### 6.6. Gait Parameters

**Figure 5:**
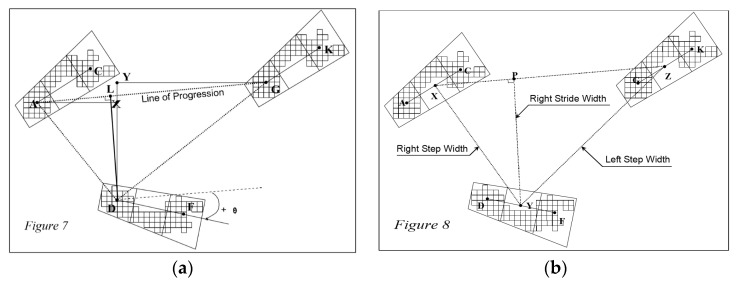
Gait Parameters in GAITRITE—definition of terms. (**a**): AG—Stride length of the left foot, AL—Step length of the right foot. (**b**): XY—right step width, YZ—left step width.

The spatiotemporal gait parameters were recorded using the GaitRite system (Instrumented walkway—version 13, CIR Systems, Franklin, NJ, USA). GaitRite is a validated tool for measuring the kinematic parameters of gait. The GaitRite System is a 20 ft electronic walkway utilized to measure the temporal (timing) and spatial (two dimensions geometric position) parameters of its pressure activated sensors. The inferred parameters are easily obtained by applying common physics and math formulas to the directly measured temporal and spatial data (i.e., calculate velocity, relationships between spatial and temporal events, etc.). The definitions of spatiotemporal parameters are as per Figure 5 from the GaitRite manual version 13 and are outlined below.

### 6.7. Spatial Parameters

Heel Centre Points (A), (D), and (G) are the heel centres of each footprint. Stride Length is the distance between the heel points of two consecutive footprints of the same foot measured on the line of progression. Step Length is the distance from the heel centre of the current footprint to the heel centre of the previous footprint on the opposite foot. In Figure 5, the length of line (AL) is the step length of the right foot, while the length of line (LG) is the step length of the second left foot. Spatial parameters are measured in centimetres (from the GaitRite manual).

### 6.8. Temporal Parameters

Stride time—time elapsed between the first contacts of two consecutive footfalls of the same foot. Step time—time elapsed from first contact of one foot to first contact of the opposite foot. Gait cycle time—elapsed time between first contacts of two consecutive footfalls of the same foot. Velocity—gait velocity was measured as metres/second. Single Support—the time elapsed the last contact of the current footfall to the first contact of the next footfall of the same foot. Double support—two periods when the both feet are on the floor initial double support and terminal double support. Swing time—is initiated with toe off and ends with heel strike. It is the time elapsed between the last contact of the current footfall and the first contact of the next footfall on the same foot (from the GaitRite manual).

### 6.9. Intervention

The patients were injected with botulinum toxin (up to 300 units of BT, Allergan, 1 vial reconstituted with 2 mL of normal saline) into the dystonic muscles in the foot. The dystonic muscles were identified clinically form dystonic posturing of the foot from repeated observation of the walking and were confirmed by DANTEC CLAVIS electromyograph/stimulation recorder.. The maximum dose of Botox used was 300 units. The dose was dependent on the number of muscles and the size of the muscle involved. The typical dystonic muscles were Extensor Hallucis Longus causing striatal big toe, Gastrocnemius/Soleus-causing equinus (plantarflexion) deformity, Tibialis Posterior causing equinovarus deformity, Flexor Pollicis Longus and Flexor Digitorum Longus causing clawing of great toe and other toes, and the intrinsic muscles of the foot. Patient specific dosage was calculated after identifying the dystonic muscles. All patients had similar input from the physiotherapist regarding stretching and strengthening of their foot muscles.

### 6.10. Data Analysis

Spatiotemporal gait parameters captured by the GaitRite Walkway System were gathered and transferred into Excel spreadsheets. Data were double checked and transferred into the Stata statistical software package (Version 13). Statistical analysis was conducted using the Stata statistical software package (Version 13). The data represented spatiotemporal gait parameter measurements on the affected leg, and the unaffected leg at two time points, before and after intervention (injection of botulinum toxin). Individual gait parameters were checked for changes over time using random effects panel data models, to allow adjustment for sex, age, and side of abnormality. Hausman tests were used to establish the validity of the random effects’ models. Also, the difference between sides was tested for a time effect in a similar model. The lower limb functional parameters—FMDS, VAS, TUG, BBG, UPDRS–LL, GAS, Cadence and Gait Velocity—were analyzed in similar models. We also looked at the symmetry ratio based on the recommendations of Patterson et al. [8], and this was applied to step length, swing time, and stance time. We calculated the symmetry ratio for these parameters and tested whether there was any change over time in these ratios (i.e., from pre-treatment to post-treatment). The Gait Variability Index proposed by Gouelle et al. [12] was considered for application to this dataset.

## Figures and Tables

**Table 1:** Patient demographics, diagnosis, muscles involved and dosing of botulinum toxin (BT).

Patients	Age (Years)	Sex	Height (cms)	Weight (kgs)	Side	Diagnosis	Muscles Involved	Botulinum Toxin Units
1	65	M	172	63	Right	PDDBS	FHL, FDL	200
2	64	F	167	65	Right	PDDBS	FHL, FDL	200
3	68	M	180	85	Right	PDDBS	FHL, FDL, FDB	200
4	48	M	165	78	Left	PDDBS	EHL	100
5	70	F	165	73	Right	PDDBS	G/S, TP	300
6	61	F	164	70	Left	FD	FHL, FDL	200
7	43	M	175	82	Left	PD	FHL, FDL	300
8	66	M	164	68	Left	PD	G/S, PL	200
9	85	F	160	59	Right	PD	EHL	100
10	72	M	178	83	Left	FD	G/S	200
11	62	M	175	75	Left	PD	FHL, FDL	200
12	76	M	170	74	Left	PD	FHL, FDL	200
13	70	M	177	83	Right	FD	G/S	200
14	75	F	185	74	Left	FD	FDL	200

FHL—Flexor Hallucis Longus, FDL—Flexor Digitorum Longus, FDB—Flexor Digitorum Brevis, EHL—Extensor Hallucis Longus, G/S—Gastrocnemius/Soleus, TP—Tibialis Posterior, PL—Peroneus Longus, PDDBS—Parkinson’s Disease with Deep Brain Stimulation, PD—Parkinson’s Disease, FD—Foot Dystonia.

**Table 2:** Gait analysis in foot dystonia pre and post injection of BT.

Gait Parameters	Side	Before Mean	SE	After Mean	SE	Average Change	SE
Stride length	Abnormal	95.4	5.6	99.9	6.2	4.5	1.9
	Normal	95.0	5.6	100.3	6.3	5.4	2.1
Step length	Abnormal	48.2	3.1	50.0	3.1	1.9	0.9
	Normal	46.7	2.8	49.7	3.3	3.1	1.7
Step differential	Abnormal	1.8	0.5	4.4	1.1	2.5	1.1
	Normal	7.2	2.5	4.2	1.6	−3.0	3.9
Gait velocity		83.4	6.4	92.0	6.6	8.6	3.9
